# Analysis of the levels of hope and influencing factors in infertile women with first-time and repeated IVF-ET cycles

**DOI:** 10.1186/s12978-021-01248-y

**Published:** 2021-10-09

**Authors:** Ying Ni, Limin Huang, Chenye Tong, Wen Qian, Qiong Fang

**Affiliations:** 1 Department of Nursing, Ruijin Hospital, Shanghai Jiaotong University School of Medicine, Shanghai, China; 2 Reproductive Medical Center, Ruijin Hospital, Shanghai Jiaotong University School of Medicine, Shanghai, China; 3 School of Nursing, Shanghai Jiaotong University, Shanghai, China

**Keywords:** In vitro fertilization-embryo transfer, Repeated cycles, Hope level, Influencing factors

## Abstract

**Purpose:**

To explore the hope levels and influencing factors in infertile women undergoing first-time and repeated in vitro fertilization-embryo transfer (IVF-ET) cycles.

**Methods:**

This study was a cross-sectional and convenient sampling study conducted among patients undergoing IVF-ET from January to June 2019. Patients were divided into first-time and repeated groups by the number of IVF-ET cycles, and then a questionnaire survey was administered. The questionnaire included demographic information, Herth hope index (HHI) scale, Locke-Wallace short marital-adjustment test scale and social support rating scale. Multiple linear regression was used to analyse the influencing factors associated with hope levels.

**Results:**

A total of 298 IVF-ET patients were recruited for the study, including 150 (50.3%) in the first-time cycle group and 148 (49.7%) in the repeated cycle group. The HHI score of the repeated cycle group was significantly lower than that of the first-time cycle group (34.4 ± 3.5 vs*.* 37.5 ± 3.7, *P* < 0.001). Multiple linear regression analysis indicated that repeated IVF-ET and age were independently negatively correlated with HHI, with standardized coefficient β values of − 0.895 and − 0.223, respectively (both *P* < 0.001). High education level (*P* = 0.002), high monthly income (*P* = 0.020), high degree of short marital-adjustment test (*P* < 0.001) and social support rating (*P* < 0.001) were independently positively correlated with HHI.

**Conclusion:**

Infertile women undergoing repeated IVF-ET have low hope levels. Maintaining a good marriage adjustment and establishing a good social support and relationship network could effectively improve their hope levels.

## Plain language summary

Hope level is closely related to patients' mental health and emotional state. Therefore, improving the hope level of infertile women with IVF-ET could contribute to relieving the psychological pressure of patients and improving the success rate of pregnancy. This study aimed to evaluate the hope level of infertile women with different IVF-ET cycles and analyse its influencing factors to provide guidance for clinical nurses to clarify the intervention objects, select reasonable intervention measures and improve the hope levels of patients. A total of 298 IVF-ET patients were recruited in the study, including 150 in the first-time cycle group and 148 in the repeated cycle group. The results showed that the hope level of patients with repeated IVF-ET cycles was significantly lower than that of patients undergoing first-time treatment. We also analysed its influencing factors and found that repeated IVF-ET cycles and age were independent negative correlation factors in hope levels. High monthly income, living in the city, high marital adjustment and social support score were independent positive correlation factors in hope levels. Therefore, maintaining a good marriage adjustment and establishing a good social support and relationship network could effectively improve their hope levels.

## Introduction

With changes in the living environment, the accelerating pace of life and delays in marriage and childbearing, the incidence of infertility is rising year by year. According to statistics from the World Health Organization (WHO), approximately 8–12% [1] of couples worldwide have experienced infertility. As consistently demonstrated, infertility has a strong and negative impact in several areas of the individual’s life. For instance, impairments in marital relationships [2, 3], sexual satisfaction [4, 5], psychosocial well-being [6–8] and psychological correlates [9, 10] are significantly associated with infertility.

Since the approaches of controlled ovarian stimulation (COS) and the conditions of the embryonic laboratory have improved, in vitro fertilization-embryo transfer (IVF-ET), as the main assisted reproductive therapy for infertile patients, has brought hope to many infertile patients. However, due to the long treatment cycle, frequent visits, high costs, various traumatic operations and uncertain results, IVF-ET is a process with hope and disappointment, exerting brings both physical and psychological pressure on infertile women [11].

Some patients experience repeated implantation failure, and approximately 25% had already undergone more than five IVF-ET cycles [12]. Repeated IVF-ET significantly increases the risk of ovarian hyperstimulation syndrome, premature delivery and oestrogen-dependent tumours [41–43]. The study also found that anxiety and stress are present in women throughout the treatment [13]. At the same time, the negative emotions caused by repeated IVF-ET failure can further reduce the success rate of pregnancy [14].

Hope is a valuable human response that has received increasing attention in recent years [15, 16]. Hope is a type of multidimensional positive life power and provides an optimistic expectation of a wonderful outcome and an effective adjustment mechanism that enables individuals to overcome current difficulties [17]. Hope can change the experiences of patients with chronic diseases and their lifestyles [18]. Hope has a beneficial effect on individuals’ health because it enables them to cope with conflicts, achieve healthy objectives, maintain quality of life, and promote health [19, 20]. Rahimipour et al. (2015) conducted a clinical trial on the effect of hope therapy on anxiety, depression and stress among 50 patients undergoing hemodialysis [21]. They found a significant reduction in mean scores of anxiety, depression and stress in the hope therapy group before and after the intervention, and changes in mean scores of depression, anxiety and stress were lower in the hope therapy group than in the placebo group. Hope level is closely related to patients' mental health and emotional state. Therefore, improving the hope level of infertile women with IVF-ET could contribute to relieving the psychological pressure of patients and improving the success rate of pregnancy. In the current literature, exploration of hope and its role among infertile women with IVF-ET has not been widely undertaken in this respect. This study aimed to use the Hertz hope index (HHI) [23] to evaluate the hope levels of infertile women with different IVF-ET cycles and analyse its influencing factors to provide guidance for clinical nurses to clarify the intervention objects, select reasonable intervention measures and improve the hope levels of patients.

## Methods

### Ethics statement

The study protocol was in accordance with ethical standards and was approved by the Ethics Committee of Shanghai Ruijin Hospital. Written informed consent was obtained from each participant. Information collected from all participants was kept confidential and anonymous.

### Data and study design

This study was a cross-sectional and convenient sampling study conducted among outpatient women diagnosed with infertility and treated with IVF-ET cycles from January to June 2019. All of the participants were recruited at the Reproductive Medical Center, Ruijin Hospital, Shanghai Jiaotong University School of Medicine in China. The inclusion criteria were as follows: ① female outpatients diagnosed with infertility and ≥ 1 IVF-ET cycle; ② women who signed informed consent forms after completely comprehending the content of the study; and ③ women who had the basic ability to read, communicate, and complete the questionnaire independently. The exclusion criteria were as follows: ① women diagnosed with previous or current mental disorders, cognitive impairment, or inability to understand the content of the questionnaire; ② women diagnosed with severe chronic diseases; and ③ women who had the embryos from previous cycles successfully implanted. The participants were divided into two groups according to the number of IVF-ET cycles. In the first-time group, participants underwent the first IVF-ET cycle. In the repeated group, each participant underwent 2 or more IVF-ET cycles. The investigation was conducted during the last follow-up period before the next cycle. After written informed consent was obtained for this study, a self-report questionnaire was distributed to each eligible participant, and clinical data were collected from their medical records. Questionnaires with any missing data were excluded from statistical analyses. All questionnaires were required to be completed independently by the patients. The questionnaires were collected, and their completeness was checked at that time. The investigation process was conducted anonymously. The questionnaire elimination criteria were as follows: ① the same answer for each question; or ② withdrawal from this research for various reasons.

The sample size estimations were performed using the formula n = Z_1−α/2_^2^ * SD^2^/d^2^ [22] with a 95% confidence interval. Based on a published study [26], we chose 5.57 as the standard deviation of the hope level within patients undergoing IVF treatment and 1.0 for the accepted margin of error. It was estimated from the sample size calculation that 240 participants (120 in each group) would meet the requirement. Considering the chances of a 20% dropout rate, we decided to recruit a minimum of 290 participants (n = 145 in each group).

A total of 315 questionnaires were distributed, and all of them were received, for a response rate of 100%. Seventeen questionnaires were excluded: 2 for missing answers, 10 for the same answers for each question, and 5 for deviation from the inclusion criteria. In total, 298 complete responses were received in the present study. The effective rate of the questionnaires was 94.6%.

### Measures

#### Demographic characteristics

The demographic characteristics were collected with a general information questionnaire designed by our panel, which included age, marriage age, residence, occupation, education level, monthly household income, and number of treatment cycles.

#### Measurement of hope level

This study used the Herth hope index (HHI) scale [23] to measure the hope level. The HHI scale consists of three dimensions: a positive attitude towards reality and the future (including Items 1, 2, 6 and 11); taking positive action (including Items 4, 7, 10 and 12); and maintaining a close relationship with others (including Items 3, 5, 8 and 9). All items were scored using a 4-level Likert scoring method with 1–4 points representing very opposed, opposed, agree and very much agree, respectively. The total score of the scale is 12–48 points, among which the low, medium and high hope levels are 12–23 points, 24–35 points and 36–48 points, respectively. The higher that the score is, the higher that the level of hope is of the respondent. The Cronbach's α value of the reliability of the scale was 0.85 [23].

#### Measurement of marriage moderation

The Locke Wallace short marital adjustment test (MAT), jointly developed by Locke and Wallace, was used to assess marriage moderation. The MAT scale is designed to objectively and quantitatively assess the individual's adaptability and satisfaction with the relationship between husband and wife [24]. The scale consists of 15 items, reflecting the adjustment of marriage from 15 aspects. The total score ranges from 2 to 158. The higher that the score is, the better that the marriage adjustment is, and the higher that the quality of the marriage is. If the score is less than 100, it means that the marriage is maladjusted. If the score is more than 100, it means that the marriage has adapted well. The reliability coefficient of the questionnaire was 0.90, and the discriminant ratio was 17.5, indicating good reliability and validity.

#### Measurement of social support

The Chinese version of the Social Support Rating Scale (SSRS), designed by Xiao Shuiyuan, was used to assess social support [25]. The scale has 10 items, including objective support (3 items), subjective support (4 items) and support utilization (3 items). The total score range is 12–66. The higher that the score is, the more support that there is. The consistency of the total score was 0.92, and the consistency of each item was 0.89–0.94.

### Statistical analysis

In this study, statistical analysis was performed with SPSS software, version 23.0. The measurement data are presented as the mean ± standard deviation, and the enumeration data are expressed as the frequency and constituent ratio (%). Student’s t test was used to compare the two groups. The x^2^ test was used to test the rate inspection. Pearson’s correlation analysis (r) was used to analyse the correlations of continuous variables or rank data. Stepwise analysis was used for multivariate analysis. Retest reliability was tested by consistency κ value representation. The validity was analysed by principal component analysis, factor analysis and principal component analysis. P < 0.05 was considered statistically significant.

## Results

### Description of the participants

There were 150 patients in the first-time group out of 298 participants, while the 148 in the repeated group had 53 with two IVF-ET cycles, 51 with three cycles, and 44 with four or more cycles. The age and marriage age of the patients in the repeated group were 33.0 ± 3.9 years old and 6.7 ± 3.9 years, respectively, which were significantly higher than those in the first-time group (31.9 ± 4.3 years old and 5.8 ± 3.9 years, respectively) (P < 0.001). The results showed that the age of patients with repeated IVF-ET was higher and the marriage age was old. Conversely, comparing the two groups, there were no significant differences in education level, permanent residence, occupation or family monthly income between the two groups (P > 0.05); see Table 1.

**Table 1:** Demographic characteristics of infertile women undergoing IVF-ET with different cycles

Variables	Total (n = 298)	First-time group (n = 150)	Repeated group (n = 148)	t/χ^2^	P
Age (years)	31.9 ± 4.3	31.0 ± 4.4	33.0 ± 3.9	− 4.175	< 0.001
Marriage age (years)	5.8 ± 3.9	4.8 ± 3.7	6.7 ± 3.9	− 4.165	< 0.001
Educational level, n (%)					
Junior high school or below	56(18.8)	28(18.7)	28(18.9)	0.003	0.956
Senior high school or above	242(81.2)	122(81.3)	120(81.1)		
Residence, n (%)					
Rural	67(24.2)	36(24.2)	31(24.2)	0.118	0.731
City/town	210(75.8)	113(75.8)	97(75.8)		
Occupation, n (%)					
Physical	60(20.1)	28(18.7)	32(21.6)	0.405	0.525
Mental	238(79.9)	122(81.3)	116(78.4)		
Family monthly income (yuan), n (%)					
< 5000	112(37.6)	55(36.7)	57(38.5)	4.879	0.087
5000–10,000	75(25.2)	31(20.7)	44(29.7)		
≥ 10,000	111(37.2)	64(42.6)	47(31.8)		

### Reliability and validity of HHI scale

The reliability and validity of the HHI scale were analysed before it was used in the participants of this study. Fifty patients were retested after 2 weeks, and the consistency test results indicated good test–retest reliability with k = 0.805 (P < 0.05). The patient data were analysed by principal component analysis. The results of the suitability test showed that the KMO statistical value was 0.765 (P = 0.01), indicating that factor analysis was suitable. The correlation coefficient matrix was rotated with maximum variance to extract the factors with eigenvalues > 1. The eigenvalues of the three principal components were 2.365, 1.427 and 1.119. The cumulative variance contribution rate was 69.4%. The factor components produced by statistical analysis were consistent with the theoretical structure.

### Hope level, marriage adjustment and social support

**Fig. 1:**
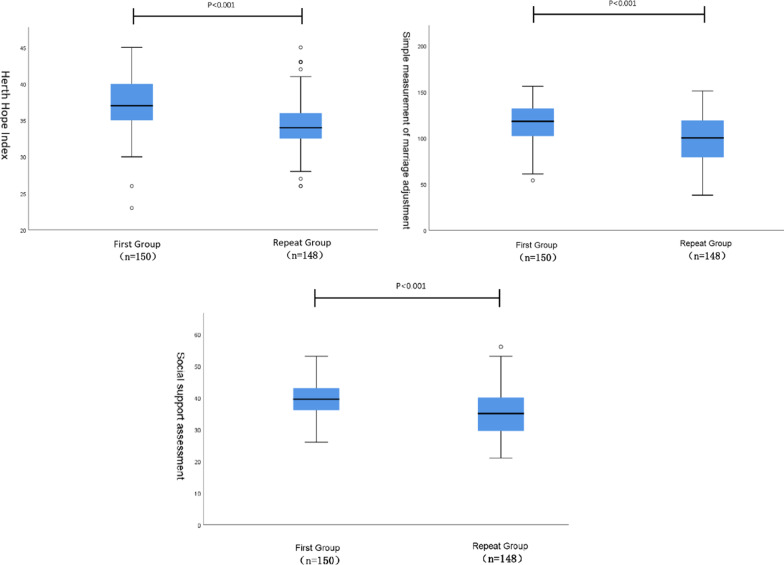
Frame chart of the differences between the two groups in Herth hope index (**A**), simple measurement of marriage adjustment (**B**), and social support assessment (**C**)

The HHI, MAT and SSRS scores in the repeated group were 34.4 ± 3.5, 99.0 ± 24.9 and 35.4 ± 7.2, respectively, which were significantly lower than 37. 5 ± 3.7, 116.3 ± 22.4 and 39.5 ± 5.2 in the first-time group (P < 0.001); see Fig. 1. The hope level, marital adjustment and social support of patients with repeated IVF-ET were significantly decreased.

Comparing the scores of the three dimensions of the HHI also showed that the scores in the repeated group were significantly lower than those of the first-time group (see Table 2). According to the scores, they were divided into low-level, medium-level and high-level subgroups. Further analysis showed that 67.3% (n = 101) of the patients in the first-time group had high levels. However, the high level was only 29.7% (n = 44) in the repeated group (P < 0.001). More than half of the patients undergoing first-time IVF-ET were at a high hope level, while the proportion of patients with a high hope level in the repeated group was significantly decreased, as shown in Table 2.

**Table 2:** HHI score in dimensions and subgroups of infertile women undergoing IVF-ET

	Total (n = 298)	First-time group (n = 150)	Repeated group (n = 148)	t/χ^2^	P
Herth hope index total score	35.9 ± 3.9	37.5 ± 3.7	34.4 ± 3.5	7.391	< 0.001
Positive attitude towards reality and future	11.9 ± 1.4	12.3 ± 1.4	11.5 ± 1.4	4.930	< 0.001
Positive actions taken	11.7 ± 1.6	12.1 ± 1.6	11.3 ± 1.4	4.373	< 0.001
Keep a close relationship with others	12.3 ± 1.9	13.1 ± 1.7	11.5 ± 1.7	7.761	< 0.001
Low level n(%)	1 (0.3%)	1 (0.7%)	0 (0.0%)	45.521	< 0.001*
Medium level n(%)	152 (51.0%)	48 (32.0%)	104 (70.3%)		
High level n(%)	145 (48.7%)	101 (67.3%)	44 (29.7%)		

*P value is the result of likelihood ratio

### Correlation analysis of influencing factors of hope level

**Fig. 2:**
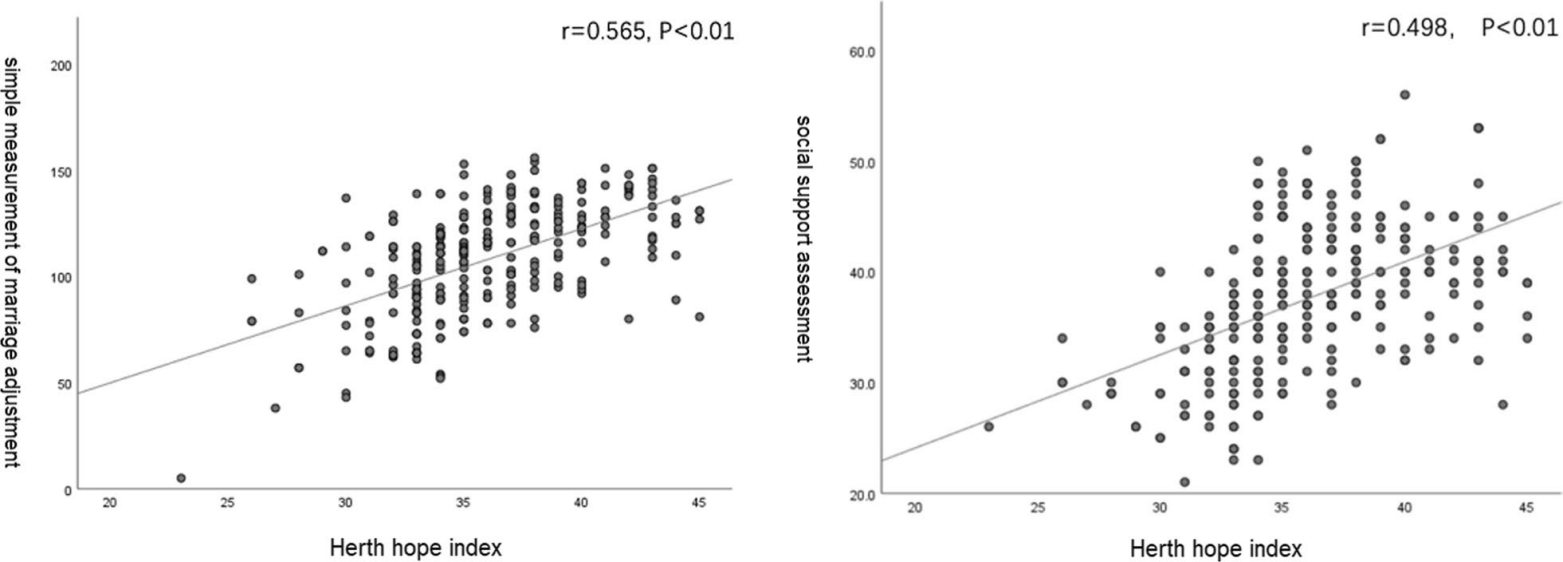
Correlation analysis of HHI with MAT and SSRS

The correlations between of the HHI with demographic characteristics, MAT and SSRS were analysed. The results showed that the HHI was positively correlated with MAT (r = 0.565, P < 0.01) (see Fig. 2a), SSRs (r = 0.498, P < 0.01) (see Fig. 2b), monthly income (r = 0.269, P < 0.01), education level (r = 0.292, P < 0.01), permanent residence (r = 0.189, P < 0.01) and mental work (r = 0.179, P < 0.01). Conversely, the HHI was negatively correlated with age (r = − 0.204, P < 0.01), marital age (r = − 0.126, P = 0.029) and repeated IVF-ET (r = − 0.480, P < 0.01). It was indicated that the hope level of older, long-term married patients and cases with repeated IVF-ET treatment was low.

### Multiple linear regression analysis on the influencing factors of hope level

HHI was used as the dependent variable, and the variables with statistical significance in univariate analysis were included in the multiple linear regression model (stepwise regression). As shown in Table 3, the results showed that repeated IVF-ET (β = − 0.859, P < 0.001) and age (β = − 0.223, P < 0.001) was negatively correlated with hope levels. However, education level, monthly income, marital age, and MAT and SSRS scores were positively correlated with the HHI. Repeated IVF-ET and age were independent factors of HHI downregulation.

**Table 3:** Multivariate linear regression analysis of influencing factors of HHI in infertile women undergoing IVF-ET

Variables	Non standardized coefficient B	Standard error	Standardized coefficient β	t	P	95% Confidence interval
Constant	30.288	2.066	–	14.664	< 0.001	26.223 ~ 34.353
Repeated IVF-ET	− 0.859	0.141	− 0 0.291	− 6.095	< 0.001	− 1.136 ~ − 0.581
MAT	0.045	0.009	0.288	5.266	< 0.001	0.028 ~ 0.062
SSRS	0.117	0.031	0.198	3.802	< 0.001	0.057 ~ 0.178
Age	− 0.223	0.059	− 0.242	− 3.779	< 0.001	− 0.339 ~ -0.107
Marriage age	0.275	0.064	0.274	4.306	< 0.001	0.150 ~ 0.401
Monthly income	0.516	0.220	0.114	2.349	0.020	0.084 ~ 0.949
Education level	1.474	0.478	0.147	3.081	0.002	0.532 ~ 2.416

R^2^ = 0.487, adjusted R2 = 0.475, P < 0.05

## Discussion

Hope is an individual's confidence and expectations to solve problems when facing difficulties. Patients with a high hope level can actively seek help and solve problems. Hope is believed to be a psychological force guiding patients to manage diseases more actively [26]. Currently, psychological research on infertile women with IVF-ET mainly focuses on anxiety, depression and stress. However, to the best of our knowledge, there has been little research focused on their hope levels. Therefore, this study conducted a cross-sectional study on the hope level and its influencing factors in infertile women undergoing IVF-ET. This study found that the HHI scores of patients with repeated IVF-ET cycles were significantly lower than that of those with first-time treatment (32.0 ± 3.3 vs. 36.0 ± 3.1, P < 0.001). The scores of positive attitudes towards reality and the future, positive action taken and close relationships with others all decreased remarkably. In the repeated group, the proportion of the high-level subgroup was only 11%, which was significantly lower than the 58.5% in the first-time group. Multivariate analysis showed that age and repeated IVF-ET cycles were independent negative correlation factors of hope level. High monthly income, living in the city, high marital adjustment and high social support score were independent positive correlation factors with hope level. The hope level score obtained by this research was lower than that obtained by TANG Nan et al. [26]. The reason could be that more infertile women with repeated IVF-ET cycles were included in this study. Among them, patients with more than 4 cycles accounted for 12.7%, and the score of these patients was significantly lower than that in the first-time group. We found that infertile women with repeated IVF-ET cycles were the main objects of nursing interventions. Maintaining good marital adjustment and establishing good social support and relationship networks might be effective ways to improve the hope levels of infertile women with IVF-ET.

In our previous study [27], we found that the psychological pressure of infertile women undergoing IVF-ET mainly comes from worries about age and pregnancy success rates, family, society and the surrounding environment. Therefore, we not only evaluated the level of hope but also conducted a targeted questionnaire survey on marriage adjustment and social support. Some studies have found that hope is positively correlated with happiness, persistence, health, social support, improvement of mental pressure and other factors [28]. Hope is an important factor in helping patients to recover when they feel stressed. This study also found that patients undergoing first-time IVF-ET had a better level of hope. These patients had clear treatment plans and great expectations for success. However, once the first cycle fails, the psychological pressure of patients increases significantly, leading to a significant decline in the level of hope.

A study from Israel found that, although the overall success rate of IVF-ET reached 54%, approximately 25% of the patients underwent more than five treatment cycles, and 12% of the patients underwent more than seven cycles [12]. Some studies have indicated that ovarian reserve function decreased and the risk of women reaching menopausal transition or menopause ahead of time increased significantly during repeated IVF-ET cycles, especially more than three cycles [44, 45]. Repeated IVF-ET failure easily causes anxiety, depression and other adverse psychological problems [29]. This study also found that repeated IVF-ET cycles were an independent negative correlation factor for the decline in hope level. Therefore, in nursing care, patients must have an objective understanding of the outcome of repeated IVF-ET cycles. Nursing staff must provide appropriate guidance and suggestions, offer more disease-related knowledge, and prevent patients from having unrealistic expectations about treatment. Based on the three dimensions of hope levels, there is one factor of "keeping close with others", in addition to other personal factors. It is easy to find the status of social and marital support of patients with the help of the HHI scale. It is also recommended that reproductive clinics guide patients to obtain more support from spouses, friends, family and so on.

This study also found that the age of patients in the repeated group was significantly higher than that in the first-time group. This finding showed that elderly patients might experience more IVF-ET cycles to obtain clinical pregnancy. The success rate of older pregnant women with first-time IVF-EF is low due to decreases in poor endometrial receptivity, immune dysfunction, embryo defects and other factors [30]. Clinical studies have found that the more that the age increases – 40 and over – the more that the FSH levels increase, and the more that the quality of embryos decreases (Aneuploidy) [31]. Long marital age is also an obvious characteristic of the repeated group. Women with shorter marriages have closer relationships with their husbands and will receive more support from their husbands. Long-term married couples will face more psychological pressure with repeated failures in long-term treatment. Li et al. [32] found that, in 260 pairs of infertile couples treated with IVF-ET, the incidence of psychological problems, such as anxiety and depression, was significantly higher than that in normal fertile couples. Infertile women undergoing repeated IVF-ET cycles often bear the dual pressures of family and society and have varying degrees of psychological problems. Additionally, in our previous study, we found that the anxiety and depression of patients with repeated implantation failure has significantly affected fertility quality of life [33]. In the process of treatment, negative emotions often affect the outcomes of pregnancy. Appropriate psychological care can help to relieve anxiety, tension and other emotions and improve the success rate of IVF-ET treatment [34]. Therefore, with older and longer married couples, we must inform them of the adverse side effects of repeated IVF-ET treatments and perform more psychological nursing. Nursing staff can act as the mediator of patients' marriages, encourage husbands to listen more and care more about patients' state and relieve patients' anxiety and tension to improve patients' hope levels. Some studies have also found that shared decision-making interventions can improve the psychological state of patients undergoing IVF-ET, improve their hope levels and treatment compliance, and significantly improve the pregnancy rate [35]. Helping patients to establish a sense of gratitude is helpful to alleviating the anxiety of patients in the process of IVF-ET [36]. This study was only a cross-sectional analysis, and we should try more interventions in the future.

### Limitations

This study had a few limitations. First, the convenience sampling method was adopted in this study, which might have affected the representation of the results to a certain extent. Second, the HHI has been more commonly used to evaluate the hope levels of cancer patients [37]. A systematic analysis of hope scores found that, in 68 studies, the Snyder Hope scale (46%) was the most commonly used, followed by the HHI [38]. Further studies using more scales, such as the Beck Scale [39], could be helpful to evaluate the level of hope more richly. Further studies must be conducted to examine whether the results of the present study are suitable for different cultural contexts. Moreover, there could still be residual variables that were not considered. Zhou [40] measured salivary amylase levels to reflect the stress states of patients. Through objective comparison, it is speculated that a high pressure level is also related to IVF-ET failure. Therefore, it deserves to be further explored.

## Conclusion

In summary, infertile women with repeated IVF-ET have a low hope level. Maintaining a good marriage adjustment and establishing a good social support and relationship network could effectively improve their hope levels. It would be greatly appreciated if authorities were involved or some charitable funds were provide to financially support repeated IVF-ET treatments, particularly for those with low monthly incomes. As medical practitioners, we should take the initiative to undertake a variety of targeted nursing interventions to better enhance the hope level of infertile women with IVF-ET. Especially for those with repeated IVF-ET cycles or low HHI scores, we can better improve their hope levels and the success rates of assisted pregnancy by guiding them and their families, encouraging them to actively seeking more social support and receive more attention from their spouses.

## Data Availability

The datasets used and/or analysed during the current study are available from the corresponding author on reasonable request.

## Abbreviations

IVF-ET: In vitro fertilization-embryo transfer
HHI: Herth hope index
COS: Controlled ovarian stimulation
MAT: Marital adjustment test
SSRS: Social Support Rating Scale
